# Characterization of patients with brain metastases referred to palliative care

**DOI:** 10.1186/s12904-023-01320-3

**Published:** 2024-01-11

**Authors:** Rebecca A. Harrison, Michael Tang, Kaoswi Karina Shih, Maria Khan, Lily Pham, Aline Rozman De Moraes, Barbara J. O’Brien, Roland Bassett, Eduardo Bruera

**Affiliations:** 1 Division of Neurology, BC Cancer, The University of British Columbia, Vancouver, BC Canada; 2 Department of Palliative Care, Rehabilitation, and Integrative Medicine, The University of Texas MD Anderson Cancer Center, Houston, TX USA; 3 Department of Neurology, University of Maryland School or Medicine, Baltimore, MD USA; 4 Department of Neuro-Oncology, The University of Texas MD Anderson Cancer Center, Houston, TX USA; 5 Department of Biostatistics, The University of Texas MD Anderson Cancer Center, Houston, TX USA

**Keywords:** Brain metastases, Palliative care, Supportive care, End of life care

## Abstract

**Purpose:**

In this study, we aimed to assess the clinical characteristics, reasons for referral, and outcomes of patients with brain metastases (BM) referred to the supportive care center.

**Methods:**

Equal numbers of patients with melanoma, breast cancer, and lung cancer with (*N* = 90) and without (N = 90) BM were retrospectively identified from the supportive care database for study. Descriptive statistics were used to analyze demographic, disease, and clinical data. Kaplan Meier method was used to evaluate survival outcomes.

**Results:**

While physical symptom management was the most common reason for referral to supportive care for both patients with and without BM, patients with BM had significantly lower pain scores on ESAS at time of referral (*p* = 0.002). They had greater interaction with acute care in the last weeks of life, with higher rates of ICU admission, emergency room visits, and hospitalizations after initial supportive care (SC) visit. The median survival time from referral to Supportive Care Center (SCC) was 0.90 years (95% CI 0.73, 1.40) for the brain metastasis group and 1.29 years (95% CI 0.91, 2.29) for the group without BM.

**Conclusions:**

Patients with BM have shorter survival and greater interaction with acute care in the last weeks of life. This population also has distinct symptom burdens from patients without BM. Strategies to optimize integration of SC for patients with BM warrant ongoing study.

## Introduction

Brain metastases are the most common tumors of the central nervous system [1]. Increasing incidence has been attributed to both improvements in systemic therapies prolonging extra-cranial control and innovations in neuroimaging detecting brain metastases [2]. While traditionally excluded from clinical trials, the approach to these patients is changing, with new clinical and academic dedication to developing impactful therapies in this population [3, 4]. At this time however, brain metastases are incurable, with estimated overall survival ranging from 3 to 36 months [5–8]. These patients are also highly symptomatic [9], and have frequent cognitive impairment [10] that may impact decision making capacity. As such, this is a population that would be expected to distinctly benefit from dedicated palliative care. At this time, the patterns of use of palliative care and its clinical impact have received limited study and warrant further exploration.

Palliative care provides multidisciplinary symptom management for the improvement of physical symptom burden, psychosocial distress, caregiver distress, as well as assistance with the decision-making process and is most meaningful when initiated early in advanced illness. Patients who receive earlier palliative care referral have shown in multiple studies to have improved quality of life, improved mood, and less aggressive care at the end of life [11–19]. The precise definition of “early” introduction of palliative care has varied between studies, though has been shown to be within 8 to 12 weeks of a diagnosis of advanced or metastatic cancer [11, 13, 17–19]. The American Society of Clinical Oncology guidelines in 2012 supported early palliative care consult for patients with advanced cancer alongside standard oncology care within 8 weeks of diagnosis and/or for patients with life expectancy less than 24 months [20]. However, optimal timing of who should be referred can also be challenging [21] and opportunities to standardize palliative care access and timing has still been seen to be in need [14, 22, 23].

Brain metastases has also been considered to be a major criteria for referral to palliative care [21] where life expectancy has been studied and becoming better understood [24] in addition to the areas of quality of life that are often affected [25]. Despite the likely high symptom burden patients with brain metastases often experience [25], palliative care referrals have often been in the inpatient setting and late in the dying process [26–28]. The impact of these referral patterns in this distinct patient population is not well understood.

In this study, we aimed to assess the clinical characteristics, reasons for referral, and outcomes of patients with brain metastases referred to the supportive care clinic (SCC). The SCC is a clinic with board-certified palliative care physicians, as well as nurses, social workers, dieticians, therapists, pharmacists focused on providing care that addresses the physical, psychological, and spiritual suffering of patients with cancer. During the time patients in this study were evaluated, there were no standard guidelines or triggers for clinic referral. Patients were referred based on the discretion of the treating oncologist(s) based on anticipated benefit from the clinical program. The primary objective was to compare the time between SCC referral and death between patients with and without brain metastases. Secondary objectives included evaluating the time between the diagnosis of brain metastases and first referral to the SCC, comparing the reasons for referral to the SCC between patients with and without brain metastases, comparing the baseline demographic and clinical characteristics between patients with and without brain metastases, and comparing changes in functional and clinical status between the first and second SCC visits for all patients.

## Methods

Ethical approval for this retrospective cohort study was provided by the Institutional Review Board (Protocol 2021–0069) at the University of Texas MD Anderson Cancer Center. Exemption for the requirement of informed consent was provided with approval of the protocol. Study procedures were in line with guidelines set forth in the Declaration of Helsinki.

**Fig. 1:**
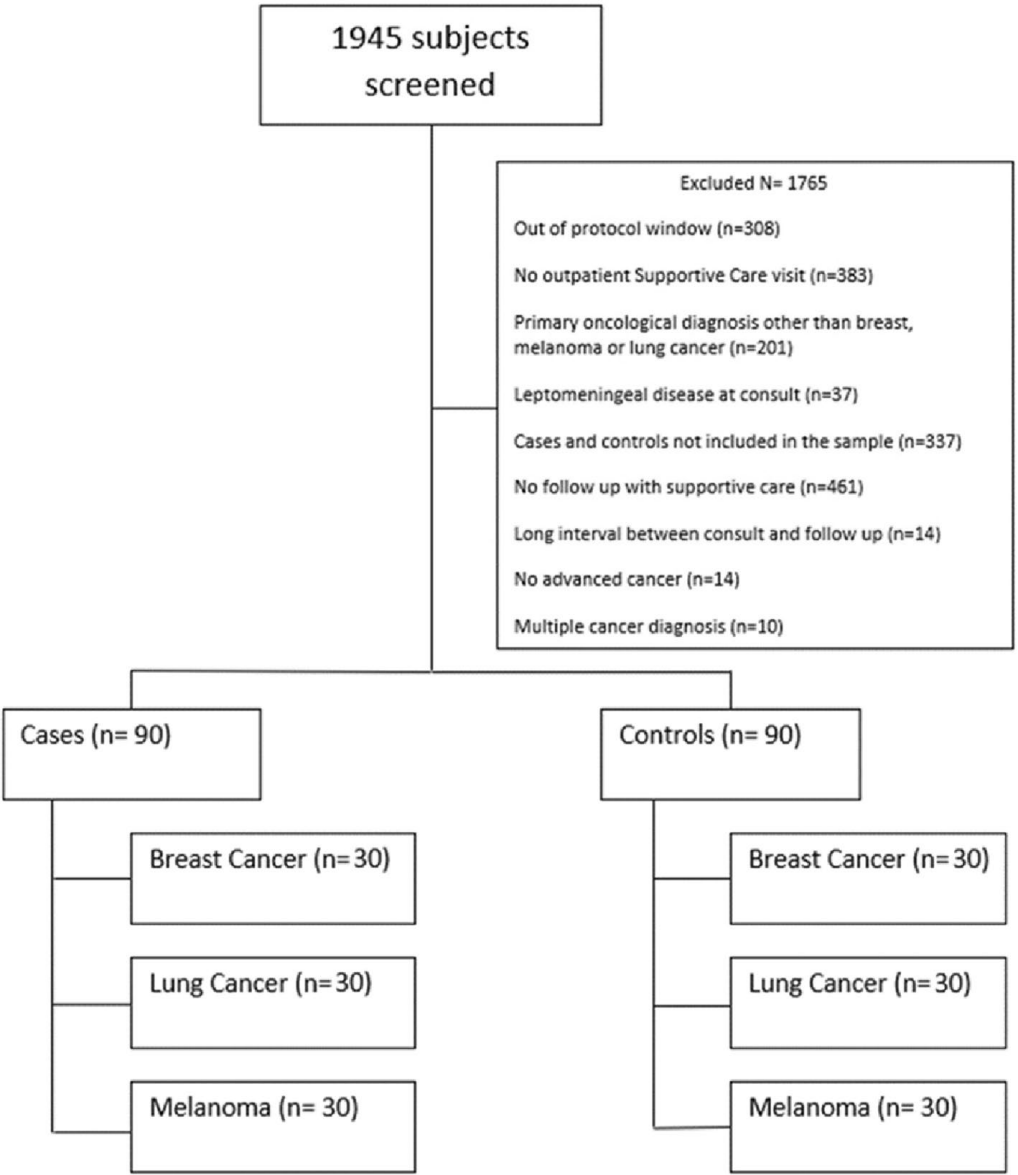
Study Flow Diagram

Patients were identified through and EPIC report generated using our inclusion criteria, and clinical data was extracted from the medical record. A total of 180 patients from 2 cohorts were analyzed: Group 1- patients with advanced cancer without known brain metastases, and Group 2- patients with advanced cancer and known brain metastases. Within each group, 30 patients were selected with each of the following cancer histologies: breast cancer, lung cancer, and melanoma. In Group 1, all patients must have had recurrent disease or locally advanced/metastatic disease (stage IIA or higher) with no known diagnosis of brain metastases at time of initial SCC visit. For Group 2, patients must have had a diagnosis of at least 1 brain metastasis at time of referral to the SCC. All patients must have had at least 1 follow up visit with SCC and been 18 years of age or older. Patients with a diagnosis of leptomeningeal dissemination at the time of their first SCC visit were excluded. A random selection of patients seen at the SCC within each histologic subgroup between March 4th, 2016 and March 4th, 2020 were included (see Fig. 1 for consort diagram).

For selected patients, demographic and disease-related data was extracted from the clinical record. Data extracted also included timing of SCC visits, health care utilization after SCC visits, Edmonton Symptom Assessment Scale scores (ESAS), Memorial Delirium Assessment Scale (MDAS) scores, CAGE-AID results, ECOG scores. Reasons for palliative care referral and documentation of advanced directives were also extracted.

Clinical and demographic characteristics were analyzed using descriptive statistics. Wilcoxon rank-sum tests were used to compare the distribution of continuous variables between patients with brain metastases and patients without brain metastases. Fisher’s exact tests were used to compare the distribution of categorical variables between groups. The method of Kaplan and Meier was used to estimate the distribution of the time between SCC referral and death. The log-rank test was used to compare distributions between groups. All statistical analyses were performed using R version 4.1.1. All statistical tests used a significance level of 5%. No adjustments for multiple testing were made.

## Results

Between March 4th, 2016 and March 4th 2020, a random selection of patients with the diagnosis of breast cancer, lung cancer, and melanoma seen at the SCC was screened (Fig. 1). A total of 1945 subjects were screened and 1765 were excluded. 90 patients were included in the brain metastasis arm (cases) and 90 patients without brain metastasis were included in the control group (controls). In both arms 30 patients were included in each of the three histologic subgroups. Table 1 shows the characteristics of enrolled patients. Patients with brain metastasis were on average younger than those without brain metastasis.

**Table 1:** Patient Characteristics (*N* = 180)

Characteristic	Brain Metastases 90 (50%)	No Brain Metastases 90 (50%)	p-value*
**Age** (in years) – median [q1-q3]	57 [47–67]	62 [54–71]	***0.01***
**Gender – n (%)**			0.87
Female	62 (68.9%)	64 (71.1%)	
Male	28 (31.1%)	26 (28.9%)	
**Ethnicity – n (%)**			0.77
Hispanic/ Latino	13 (14.4%)	12 (13.3%)	
Not Hispanic/ Latino	76 (84.4%)	75 (83.3%)	
Unknown	1 (1.1%)	3 (3.3%)	
**Race – n (%)**			0.12
African American	12 (13.3%)	7 (7.8%)	
Asian	5 (5.6%)	5 (5.6%)	
Middle East/ North African	2 (2.2%)	0	
White	68 (75.6%)	68 (75.6%)	
Other	3 (3.3%)	10 (11.1%)	
**Marital Status – n (%)**			0.33
Married	63 (70%)	60 (66.7%)	
Separated/Divorced	9 (10%)	11 (12.2%)	
Single	12 (13.3%)	7 (7.8%)	
Widowed	6 (6.7%)	12 (13.3%)	
**Education – n (%)**			0.38
Some High School	3 (3.3%)	2 (2.2%)	
Completed High School	9 (10.0%)	4 (4.4%)	
Some College	6 (6.7%)	13 (14.4%)	
Completed College	19 (21.1%)	15 (16.7%)	
Advanced Degree	10 (11.1%)	9 (10.0%)	
Unknown	43 (47.8%)	47 (52.2%)	
**Insurance – n (%)**			
Private	64 (71.1%)	65 (72.2%)	1.0
Medicare	28 (31.2%)	38 (42.2%)	0.16
Medicaid	2 (2.2%)	6 (6.7%)	0.28
Uninsured	7 (7.8%)	3 (3.3%)	0.33
**Cancer Diagnosis – n (%)**			1.0
Breast	30 (33.3%)	30 (33.3%)	
Lung	30 (33.3%)	30 (33.3%)	
Melanoma	30 (33.3%)	30 (33.3%)	
Reason for Referral To SC**			
Physical Symptom Management	81 (90%)	81 (90%)	1.0
Psychosocial Symptom Management	20 (22.2%)	23 (25.6%)	0.73
Advanced Care Planning	5 (5.6%)	4 (4.4%)	1.0
Goals of Care	4 (4.4%)	4 (4.4%)	1.0
**Referral Setting**			1.0
Inpatient	15 (16.7%)	14 (15.6%)	
Outpatient	75 (83.3%)	76 (84.4%)	
Enrolled in a Clinical Trial at time of Referral to SC**			0.75
No	59 (65.6%)	62 (68.9%)	
Yes	31 (34.4%)	28 (31.1%)	
**ECOG: Baseline**			0.79
0	5 (5.6%)	3 (3.3%)	
1	18 (20%)	17 (18.9%)	
2	34 (37.8%)	41 (45.6%)	
3	31 (34.4%)	28 (31.1%)	
4	2 (2.2%)	1 (1.1%)	

*Fisher’s exact test, ** SC- Supportive Care

Table 2 shows the intensity of symptoms based on the ESAS score at the initial consultation at the SCC and upon follow up. Those without brain metastasis tended to have higher pain scores at baseline and they tended to have worse symptoms of well-being and more problems with sleep upon follow up when compared to the brain metastasis group. When evaluating the change in total overall ESAS scores for all patients from baseline to follow up, we found a significant lower value at follow up with a mean difference change of − 3.54 (standard deviation 18.13, *p* < 0.01).

**Table 2:** ESAS-FS values at Baseline and Follow up

	Brain Metastases (*n* = 90)	No Brain Metastases (*n* = 90)	p-value*
**ESAS-FS: Baseline (median) [q1-q3]**			
Pain	4 [2.3–6.8]	6 [3.3–8]	***< 0.01***
Fatigue	5.5 [4–8]	6 [4–8]	0.56
Nausea	1 [0–4]	1 [0–5]	0.76
Depression	1 [0–4]	2 [0–4]	0.38
Anxiety	2 [0–5.8]	2 [0–5.8]	0.70
Drowsiness	3 [0–5]	3.5 [0–6]	0.46
Shortness of Breath	1 [0–5]	2 [0–4.8]	0.43
Appetite	5 [2–8]	5 [2–7]	0.93
Wellbeing	5 [3–7]	5 [3–7]	0.68
Sleep	5 [2–7]	5 [2–7]	0.80
Financial	1 [0–5]	0 [0–3]	0.06
Spiritual	0 [0–0]	0 [0–1]	0.37
Total Symptom Score	37 [25–49]	40 [28–52]	0.31
**ESAS-FS: Follow up (median) [q1-q3]**			
Pain	4 [0.25–6.8]	5 [2–7]	0.10
Fatigue	5.5 [3–8]	6 [4–7.8]	0.95
Nausea	0 [0–3.8]	1.5 [0–3]	0.57
Depression	1 [0–3]	1 [0–4]	0.83
Anxiety	2 [0–5]	2 [0–4]	0.68
Drowsiness	3 [1–3]	2.5 [0–5]	0.31
Shortness of Breath	1 [0–4]	1 [0–5]	0.21
Appetite	5 [2.3–7]	4 [2–6]	0.51
Wellbeing	4 [2–5.8]	5 [3–7]	***0.02***
Sleep	4 [1.3–6]	5 [3–7]	***0.01***
Financial	1 [0–3]	1 [0–4]	0.57
Spiritual	0 [0–1]	0 [0–1]	0.96
Total Symptom Score	32 [23–46]	37 [26–46]	0.22

*- Fisher’s exact test

Overall, advanced directives were more likely to be completed by patients without brain metastases (*N* = 64, 71.1%) than those with brain metastases (*N* = 52, 57.8%, *p* = 0.09). At baseline, 30.0% (*N* = 27) of patients with brain metastases and 27.8% (*N* = 25) of patients without brain metastases had advanced directives in place. However by the time of first follow up, a greater proportion of those without brain metastases had completed them (*N* = 51, 56.7% versus *N* = 45, 50.0%).

**Fig. 2:**
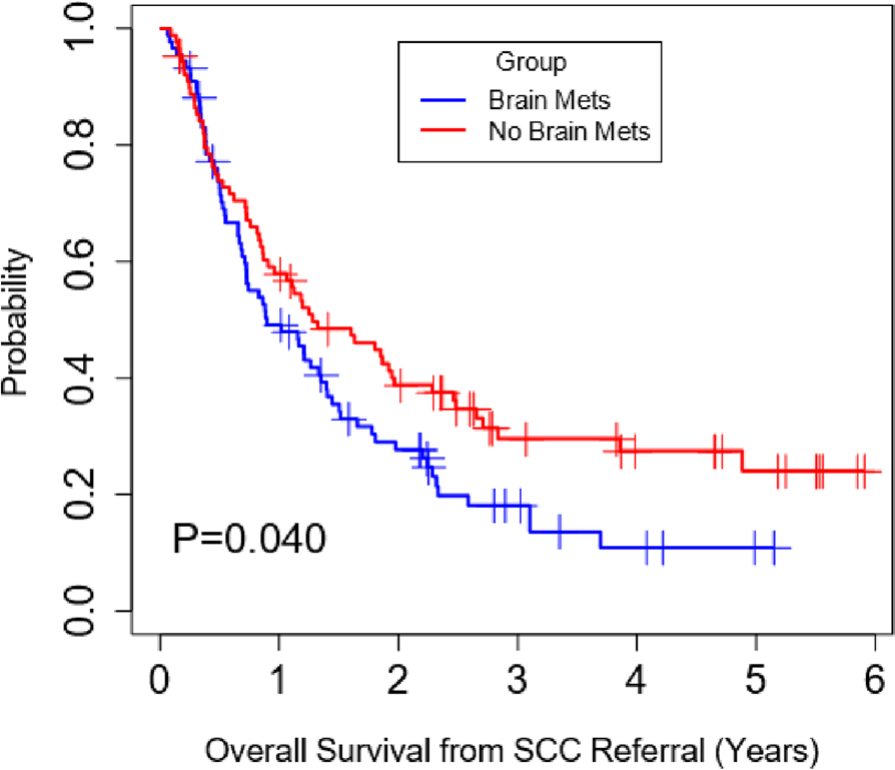
Time between SCC referral and death

The time between referral to the SCC and death can be seen in Fig. 2. The median survival time from referral to SCC was 0.90 years (95% CI 0.73, 1.40) for the brain metastasis group and 1.29 years (95% CI 0.91, 2.29) for the group without brain metastasis. The estimated overall survival at 1 year was 49.2% (95% CI 39.7, 60.9%), 18.2% (95% CI 11.1, 29.6%) at 3 years, and 10.9% (95% CI 5, 23.5%) at 5 years for those with brain metastasis. The estimated overall survival was 58% (95% CI 48.6, 69.3%) at 1 year, 29.6% (95% CI 21, 41.9%) at 3 years, and 24.1% (95% CI 15.2, 38.1%) at 5 years for those without brain metastasis.

Figure 2 shows the Kaplan-Meier plot of overall survival (OS) from the time of SCC referral by group (brain metastasis versus no brain metastasis). Patients with brain metastasis have a worse prognosis compared to patients without brain metastasis. The hazard ratio for brain metastasis versus no brain metastasis is 1.44, with a 95% confidence interval of (1.02, 2.05).

Estimates of median time from brain metastasis diagnosis to referral to the SCC can be seen in Table 3. The median time to referral after diagnosis was 0.3 years (95% CI 0.14, 0.47).

**Table 3:** Timing of Brain Metastasis diagnosis to SCC Referral

Group	N	N Events	Median^a^	Lower 95% CI	Upper 95% CI
Overall	90	90	0.30	0.14	0.47

^a^This represents median time of years to referral to the Supportive Care Center

## Discussion

This study provides new insight into the patterns of palliative care provided to patients affected by brain metastases. This is the first study to our knowledge that systematically compares patients with brain metastases to those with advanced cancer and no known CNS disease to identify distinctions in their palliative care usage and needs and their clinical trajectory after SC intervention. While the American Society for Clinical Oncology has advised that palliative care should be standard for all patients with advanced cancer [29], it is known that SC remains underutilized in oncology as a whole, and when delivered, is often done so in suboptimal fashion [30]. Through analysis of patients with brain metastases in comparison to cancer patients without CNS involvement, we were able to identify distinctions in patterns of SC usage and the clinical trajectory that ensues between these populations.

Patients with a diagnosis of brain metastases may have distinct reasons for being referred to SC and may also derive different benefits from SC. In our study, we found the most common reason for referral in both patients with and without brain metastases was physical symptom management. Studies to date, however, suggests patients with neurologic illness may have unique symptom profiles from other non-neurologic patients, with significant existential suffering, derived from the progressive disability and loss of autonomy that occurs with these illnesses [31–33]. It may be that clinicians select this reason for referral out of habit, or as other reasons for referral do not seem to capture the nature of their concern for the patient. We did find that patients with brain metastases had significantly lower pain scores on the ESAS at time of presentation to SC, suggesting pain may not have been as significant a driver for SC referral in this cohort. The similar reason for referral between these groups in our study could also reflect the patterns of practice of oncologists referring to SC. Oncologists may have established ideas regarding the role of SC in the care of cancer patients, and be more inclined to refer when patients have those symptoms traditionally considered the focus of SC, such as pain and other refractory somatic symptoms, as opposed to the other forms of suffering that are uniquely challenging in neurology patients. Increasingly, the importance of understanding how to optimally deliver SC to patients with neurologic illness is being studied. This includes optimal identification of symptoms burdens associated with CNS malignancies. While ESAS is a robustly-validated tool for all cancer types, several tools which are CNS-specific have been developed to be used in conjunction with this broader assessment tool [34, 35]. The frequent cognitive impairment in this population and higher carepartner distress also suggests further study of how to assess symptoms and suffering in this population is warranted. Dedicated evaluation in the brain metastases population may provide further insight into the potential contribution of SC to this patient group.

We found that on average, patients affected by brain metastases had shorter overall survival after their initial interaction with SC, and also had more frequent interactions with acute care. The timing of SC referral has been strongly tied to its impact on patient outcome, with earlier SC referral being associated with better quality of life and survival outcomes for patients, as well as improved health care utilization patterns at end of life [11, 36]. The largest proportion of patients in our study were referred to SC within 3 months of diagnosis with brain metastases, suggesting that it was in fact recognized these patients should be seen shortly after diagnosis with brain metastases. This is converse to literature which exists in this area, which has found late referrals amongst those that do get referred to SC are common, with most patients being referred in the last 1.6 months of life [27]. While a comprehensive view of this area would also require us to evaluate what proportion of these patients are in fact referred, this does appear better than the literature would suggest. This may be because this study was based at a quaternary referral center, where palliative care specialists are available and there is broad recognition of the discipline’s contribution to patient care. This patient population was found to have greater interactions with the acute care settings towards the end of life, with higher rates of ICU admission, emergency room visits, and hospitalizations after initial SC visit than those patients without brain metastases, events used to measure quality of end of life care per ASCO recommendations [37]. This may support the increased rates of complications and issues that arise in this population, and perhaps a need to integrate SC even earlier in the illness course. It also emphasizes the importance of addressing goals of care and advanced directives in this patient population.

Establishing goals of care and advanced directives which align with patient values is a central component of SC. The contribution of advanced care planning to medical care satisfaction and patient outcomes is increasingly recognized. It has been shown to increase patient and family satisfaction with care, improve physician and family alignment with patient wishes, and reduce chances of death in acute care settings [38–40]. The institution of advanced care planning has also been associated with reduced medical care costs towards end of life [41], highlighting their potential economic advantages. Notably, establishing goals of care can be associated with distinct challenges in the context of patients with central nervous system issues [42], where disease impact on cognition places the patient at risk for future decisional incapacity. While our population with brain metastases and those without had similar rates of advanced directive completion at time of initial SC visit, increasing disparity was noted at time of first follow up, with patients with brain metastases being less likely to have advanced directives completed than their counterparts without CNS involvement. Given the increased risk of clinical decline in this population which could impact the ability to complete these directives, dedicated effort to ensure these are completed would be beneficial in this population. A potential reason is that the development of cognitive symptoms made their completion at time of follow up more complex or time-consuming than with other patients. Early completion of advanced directives in the cancer course, even prior to the diagnosis of brain metastases, could facilitate the delivery of goal-concordant care [43]. Further study is warranted to elucidate the reasons for these not being completed in a significant proportion of this population, and strategic initiatives to facilitate advanced directive completion would be meaningful.

This study provides new insight into the care of patients with brain metastases, but does carry several limitations, including its retrospective design. This study being carried out at a quaternary cancer center may also impact the patterns of clinical care, as SC was readily available to the patients studied, which may not be the case at all centers. We also note the small proportion of our patients with Medicaid support, and the predominance of white patients in our study. These demographic patterns may influence health status and interactions with the health care system, which could influence patterns of SC usage. Because of these limitations, we encourage prospective study in larger and more heterogeneous patient cohorts to explore the findings of this hypothesis-generating study. Despite these limitations, our study provides new insight into SC usage in this patient population. With the increasing prevalence of brain metastases, an understanding of SC in this population has become increasingly valuable. Our findings support the need for further study and initiative in improving advanced directives and goals of care planning for this subpopulation of cancer patients, who are at risk for both short-term clinical and cognitive decline. Efforts to understand the elements of SC that are impactful for patients with brain metastases and how to deliver these in varied health care settings is an important future areas of study.

## Data Availability

The data underlying this article cannot be shared publicly for the privacy of those that participated in this study. Informed consent for data sharing was not obtained due to its retrospective nature, as approved by the Institutional Review Board at the University of Texas MD Anderson Cancer Center. The data will be shared on reasonable request to the corresponding author.
